# Effective Connectivity During Rest and Music Listening: An EEG Study on Parkinson’s Disease

**DOI:** 10.3389/fnagi.2021.657221

**Published:** 2021-04-28

**Authors:** Eleonora Maggioni, Federica Arienti, Stella Minella, Francesca Mameli, Linda Borellini, Martina Nigro, Filippo Cogiamanian, Anna Maria Bianchi, Sergio Cerutti, Sergio Barbieri, Paolo Brambilla, Gianluca Ardolino

**Affiliations:** ^1^Department of Neurosciences and Mental Health, Fondazione IRCCS Ca’ Granda Ospedale Maggiore Policlinico, Milan, Italy; ^2^Department of Pathophysiology and Transplantation, University of Milan, Milan, Italy; ^3^Department of Electronics, Information and Bioengineering, Politecnico di Milano, Milan, Italy

**Keywords:** Parkinson’s disease, music, EEG, brain connectivity, Granger causality analysis

## Abstract

Music-based interventions seem to enhance motor, sensory and cognitive functions in Parkinson’s disease (PD), but the underlying action mechanisms are still largely unknown. This electroencephalography (EEG) study aimed to investigate the effective connectivity patterns characterizing PD in the resting state and during music listening. EEG recordings were obtained from fourteen non-demented PD patients and 12 healthy controls, at rest and while listening to three music tracks. Theta- and alpha-band power spectral density and multivariate partial directed coherence were computed. Power and connectivity measures were compared between patients and controls in the four conditions and in music *vs.* rest. Compared to controls, patients showed enhanced theta-band power and slightly enhanced alpha-band power, but markedly reduced theta- and alpha-band interactions among EEG channels, especially concerning the information received by the right central channel. EEG power differences were partially reduced by music listening, which induced power increases in controls but not in patients. Connectivity differences were slightly compensated by music, whose effects largely depended on the track. In PD, music enhanced the frontotemporal inter-hemispheric communication. Our findings suggest that PD is characterized by enhanced activity but reduced information flow within the EEG network, being only partially normalized by music. Nevertheless, music capability to facilitate inter-hemispheric communication might underlie its beneficial effects on PD pathophysiology and should be further investigated.

## Introduction

Parkinson’s disease (PD) is a disabling neurodegenerative condition affecting 2–3% of the elderly population (Poewe et al., 2017). PD exhibits heterogeneous clinical manifestations that go beyond the cardinal motor features, including variable degrees of cognitive, autonomic and sleep alterations as well as neuropsychiatric symptoms (Greenland et al., 2019).

Loss of dopaminergic neurons and α-synuclein neuronal aggregates are recognized as key neuropathological features of PD, but the pathways of neuropathology progression from the basal ganglia to neocortical regions are still debated (Walsh and Selkoe, 2016), and a complete picture of the brain networks underlying motor and non-motor symptoms is still missing. A better knowledge of the brain functional connectivity signatures of PD is essential to predict the rate of disease progression, assess the risk of comorbidities and define innovative and more targeted treatment strategies. Among them, wider clinical consensus is being received by interventions that involve music, which seem to enhance motor, sensory and cognitive functions in PD patients (Raglio, 2015) but whose action mechanisms remain largely unknown (Devlin et al., 2019).

Electroencephalography (EEG) is a non-invasive, powerful and relatively available technique that can provide key information on the cerebral underpinnings of PD at the neuronal time scale. Although most research works focused on the nigrostriatal pathology, abnormal cortical oscillatory activity has emerged as a key pathophysiologic mechanism for PD (Singh, 2018). These alterations especially involve the motor cortex, where PD patients show lower basal EEG frequency (Soikkeli et al., 1991) and prominent beta oscillations during movement (Lindenbach and Bishop, 2013) compared to healthy subjects. Recent studies have explored the correlation between EEG variables, mainly power spectral density, and clinical domains in PD patients (Geraedts et al., 2018a,b). Although most studies reported no consistent associations between motor functioning and EEG power in the main frequency bands, a big sample study suggested a link between PD progression and EEG slowing (Morita et al., 2009). Notably, EEG slowing and higher theta power were correlated with cognitive deterioration and resulted predictive of dementia development (Klassen et al., 2011; Guner et al., 2017).

Fewer neurophysiological studies assessed the strength of functional connections among brain regions in PD, at rest (Stoffers et al., 2008; George et al., 2013; Teramoto et al., 2016; Utianski et al., 2016) or during exposure to external stimuli (Palmer et al., 2010; Tropini et al., 2011; Yuvaraj et al., 2016), leading to findings that need confirmation. In resting-state studies, excessive alpha synchronization was suggested as an early feature of PD (Stoffers et al., 2008), whereas positive associations emerged between (i) disease severity and theta/beta connectivity (Stoffers et al., 2008; George et al., 2013), (ii) executive dysfunction and left frontoparietal alpha coherence (Teramoto et al., 2016). Abnormal functional connectivity in PD has also emerged from task-based studies, but seems to strongly depend on the involved executive domain. Indeed, PD patients showed general hyper-synchronization during motor tasks (Palmer et al., 2010) but hypo-synchronization during emotional processing (Yuvaraj et al., 2016). Such opposite findings raise further questions on the different neural pathways engaged in PD patients by music, which has a strong motivational effect, triggering emotions, while facilitating rhythmic movements.

To our knowledge, no EEG studies investigated the immediate effects of music listening on brain activity and connectivity in PD subjects. This issue is particularly interesting, since in PD auditory rhythms and music may function as a template for organizing movements, helping coordination and improving attention (Calabro et al., 2019). Although many studies demonstrated that music therapy has beneficial effects on both motor and non-motor symptoms (Garcia-Casares et al., 2018; Ferreri et al., 2019), so far only one magnetoencephalography study provided preliminary evidence of its effects on auditory-motor connections in PD patients (Buard et al., 2019). EEG evidence on music processing in healthy subjects suggests that neuronal responses to music not only reflect the singular music characteristics, but can also largely vary from subject to subject as a function of their emotional involvement (Kaur et al., 2017). In this line, recent studies have proposed a multimodal EEG-based emotion classification during music listening (Koelstra et al., 2011; Lin et al., 2014). The obtained results confirm the complexity of neurophysiological and behavioral responses to music and further stress the relevance of studying music-induced EEG mechanisms in PD.

In this article, for the first time, we explored the directional EEG connectivity patterns underlying resting state and music listening in PD patients in comparison with healthy elders. We aimed to (i) characterize the basal cortico-cortical pathways of propagation of information in PD patients, (ii) assess any modifications induced by listening to different music tracks in their brain network. In view of the EEG slowing and enhanced theta power characterizing PD (Moazami-Goudarzi et al., 2008), and of the key role of theta and alpha EEG rhythms in music processing (Maity et al., 2015), we focused on the theta and alpha frequency bands. A traditional power spectral analysis was also performed to provide a clearer interpretation framework for the connectivity results.

The causal neuronal interactions among brain regions were examined through Wiener-Granger causality analysis (GCA), a powerful statistical tool for studying effective connectivity, i.e., the influence that one neural system exerts over another. GCA examines the statistical dependences between a current measurement of neuronal activity from one region and the past measurements of neuronal activity from other regions (Friston et al., 2013). GCA has provided meaningful descriptions of causal influences in the brain (Roebroeck et al., 2005; Maggioni et al., 2014) and is increasingly applied in neuroscientific research. In EEG studies, GCA provides the important advantage of studying specific frequency bands, allowing a deeper understanding of the dynamic influences of multiple cortical oscillators compared to time-domain connectivity measures.

## Materials and Methods

### Study Population

Fourteen subjects affected by PD and 12 healthy controls (HC) were enrolled in the study. Inclusion criteria for patients comprised diagnosis of idiopathic PD by using the UK Brain Bank criteria (Daniel and Lees, 1993), disease duration ≥ 2 years, medication with stable dose of anti-parkinsonian drugs for at least 4 weeks before the study, Hoehn and Yahr stage between 2 and 3. Exclusion criteria for all participants were: age ≥ 85 years, cognitive impairment (Mini-Mental State Examination (MMSE) < 26, Montreal Cognitive Assessment (MOCA) < 15), current treatment with neuroleptic drugs, epilepsyor major neurological, psychiatric or medical illnesses (other than PD in the patient group), and EEG alterations.

All subjects provided a written informed consent to the study protocol, which was approved by the Ethical Committee of the Fondazione IRCCS Ca’ Granda Ospedale Maggiore Policlinico, Milan, Italy, and conducted in accordance with the Declaration of Helsinki.

### Clinical and Cognitive Assessment

In PD patients, information on age of onset, laterality of motor symptoms, current medication and disease severity based on the Movement Disorders Society-Unified Parkinson’s Disease Rating Scale-III (MDS-UPDRS-III) were collected. In HC, the absence of neurological disorders was determined using a neurological examination. The participants’ cognitive functioning was rated using MMSE and MOCA. Since levodopa can modify cognition and cortical activity in PD (Poletti and Bonuccelli, 2013; Melgari et al., 2014), neuropsychological testing and EEG were performed between 60 and 90 min after its administration.

### EEG Acquisition

EEG recordings were obtained using a Micromed System Plus (Micromed S.p.A., Treviso, Italy) equipped with 8 electrodes (Fp2-C4-T4-O2-Fp1-C3-T3-O1) distributed according to the international 10–20 system and 1 electrocardiographic electrode. EEG signals were sampled at 256 Hz, using a monopolar montage with a common reference. All electrode impedances were kept below 20 kΩ.

During the EEG session, the participants were sitting in a dimly lit room with their eyes closed. The experiment consisted of a 5 min resting-state period followed by the listening to three musical pieces, (1) Bach’s keyboard concerto in D minor, BWV1052: I Allegro (Baroque style), (2) Mozart’s Piano Concerto No. 21 in C major, K467, “Elvira Madigan”: II. Andante (Classical style), (3) Dona Dona Yiddish song by Sholom Secunda and Aaron Zeitlin (*canzone* style). The choice of three different music styles was motivated by knowledge of music-specific effects on brain function and by the ultimate interest in assessing music-specific therapeutic potentials for PD. We thus adopted an exploratory design and compared a classical Mozart piece, a Bach theme with enhanced polyphonic complexity, and a traditional ballad with higher groove and dance entrainment. Notably, the Dona was included in a music-based motor therapy program for PD undertaken in our institute. The three tracks were administered in a randomized order to minimize their mutual influence in the group-level statistics. The Bach EEG recording from one HC was excluded from the analyses because it was erroneously recorded with a different montage.

### EEG Processing

The EEG data processing was performed using Brain Vision Analyzer 2.2 (BVA, Brain Products, Gilching, Germany) and Matlab R2019a (the Mathworks, Inc.). In BVA, raw EEG signals were subjected to a 2nd order zero phase Butterworth infinite impulse response filter with 0.5–70 Hz bandpass and 50 Hz notch. The EEG signals were then visually inspected and artefactual intervals were manually marked. EEG data were then imported into Matlab R2019a. Using in-house scripts, each dataset was segmented from 60 to 90 s with respect to the start acquisition time. In the music tracks, this interval was homogeneous in terms of sound and instrumental components. The interval was further segmented into consecutive, non-overlapping 2 s epochs that were subjected to the power and connectivity analyses. Artefactual epochs were excluded, while making sure that not less than 8 epochs per dataset were kept; this condition was always satisfied.

#### Power Spectral Density Analysis

For each EEG epoch, we computed the fast Fourier transform (FFT) algorithm and obtained the power spectral density (PSD). EEG power in theta (4–8 Hz) and alpha (8–13 Hz) bands was estimated using a trapezoidal numerical integration in the corresponding frequency range. The approximate EEG power integral from 0 Hz to the Nyquist frequency was used as reference to extract the relative PSD (rPSD) in theta and alpha bands. For each subject, task condition and frequency band, the resulting rPSD estimates were averaged across time epochs. To measure the rPSD changes induced by music compared to rest, normalized rPSD measures were computed as follows:

(1) $$r\,P\,S\,D_{\%change}=\frac{\left(r\,P\,S\,D_{m\,u\,s\,i\,c}-r\,P\,S\,D_{r\,e\,s\,t}\right)}{r\,P\,S\,D_{r\,e\,s\,t}}\cdot 100$$

#### Granger Causality Analysis

For each EEG epoch, the effective connectivity among the 8 EEG nodes was estimated through GCA. By Granger’s definition, a time series *X_t* is said to cause another time series *Y_t* if the latter is better predicted using all available information than if the information apart from *X_t* had been used (Granger, 1969), that is, *X_t* Granger-causes *Y_t* if the knowledge of *X_t* past significantly improves *Y_t* prediction. Assuming stochastic and wide-sense stationary processes, GCA can be applied using multivariate autoregressive (MVAR) models.

Here, GCA was performed by adapting functions from the Matlab GMAC toolbox^1^ (Tana et al., 2012). A 10th order MVAR model was fitted to the 8 EEG signals, epoch by epoch. This order resulted from the averaging of optimal orders based on Bayesian Information Criterion across epochs, conditions, and subjects. The MVAR model coefficients were then used to make inference on the information flow within the network using the Partial Directed Coherence (PDC). For each couple of nodes *i* and *j*, PDC measures the intensity of information from node *i* to node *j* and *viceversa* in a selected frequency. We obtained PDC adjacency matrices for the theta and alpha bands by summing the PDC values in the corresponding frequencies. The significance against the null hypothesis of no causality was assessed using a phase randomization surrogate test; the PDC values of the real EEG time series were compared to those of 200 phase randomized surrogate time series using a one-sided *t*-test (*p* = 0.05, Bonferroni’s corrected with *n* = 56, i.e., number of pairwise node combinations) and all non-significant connections were set to zero. The resulting adjacency matrix was normalized with respect to the maximum. Node-level PDC measures were extracted by summing the strength of incoming and outgoing information. The PDC matrices were averaged across epochs, obtaining one adjacency matrix per subject, condition and band. PDC changes from rest to music listening (PDC_%__change_) were extracted as in Eq. (1).

### Statistical Analyses

Group comparisons in terms of demographic and cognitive variables were made using two-sample *t*-tests and Fisher’s exact tests for continuous and categorical data, respectively (*p* < 0.05). In EEG power analysis, the channel-level theta/alpha rPSD values during rest and music conditions were compared between PD and HC using non-parametric two-sided Wilcoxon ranksum tests. These tests were also performed on channel-level theta/alpha rPSD_%__change_ from rest to music to inform on possible differences between PD and HC in terms of music effects on EEG power. In all group comparisons, the significance level was placed at *p* = 0.05, uncorrected and after Bonferroni’s correction with *n* = 8, number of EEG nodes. In each group, the significance of rPSD changes from rest to music was assessed through a Wilcoxon signed rank test (*p* < 0.05). In GCA—for each group, condition, and band—we assessed the ranking of the network nodes in terms of PDC inflow/outflow through Kruskal-Wallis tests. If significant differences among network nodes emerged (*p* < 0.05), *post-hoc* pairwise comparisons were performed (*p* < 0.05). This analysis allowed to identify the main “drivers” and “receivers” of information within the EEG network. In each group, the significance of PDC changes from rest to music was assessed through a Wilcoxon signed rank test (*p* < 0.05).

For each condition and frequency band, link-, node-, and network-level PDC values were compared between PD and HC using non-parametric two-sided Wilcoxon ranksum tests. Significance was set to *p* < 0.05; in the link- and node-level tests, a Bonferroni’s correction was applied with *n* = 56 and 8, respectively. To assess group differences in the PDC changes induced by music listening, the same tests were applied to PDC_%__change_ values (*p* < 0.05).

## Results

### Demographic, Clinical, and Cognitive Information

The sample characteristics are reported in Table 1. No significant differences in terms of age, sex, educational attainment, dominant hand, and cognitive functioning based on MOCA and MMSE emerged between PD patients and HC.

**TABLE 1:** Demographic and clinical information of the sample.

	**PD (*n* = 14)**	**HC (*n* = 12)**	**Test**	***p***
Age (years)	69.36 ± 4.54	69.25 ± 6.26	*T* = 0.962	n.s.
Gender (male/female)	6/8	6/6	*h*_Fisher_ = 0	n.s.
Years of education	13.57 ± 3.56	13.38 ± 3.90	*T* = 0.896	n.s.
Handedness (left/right)	0/14	1/11	*h*_Fisher_ = 0	n.s.
MMSE	28,12 ± 1.41	28.3 ± 1.22	*T* = 0.733	n.s.
MOCA	24.40 ± 2.90	25.76 ± 0.90	*T* = 0.132	n.s.
Age of onset (years)	60.92 ± 8.01	n.a.	n.a.	n.a.
Side dominance of motor symptoms (left/right)	7/7	n.a.	n.a.	n.a.
Levodopa equivalent daily dose (mg)	642.14 ± 293.91	n.a.	n.a.	n.a.
MDS-UPDRS III	25.43 (±5.12)	n.a.	n.a.	n.a.

*For continuous variables, mean ± standard deviations are reported. MMSE, Mini-Mental State Examination; MOCA, Montreal Cognitive Assessment; MDS-UPDRS III, Movement Disorder Society—Unified Parkinson’s Disease Rating Scale III; T, T-statistics; h_Fisher_, Fisher’s exact test decision; n.a., not applicable; n.s., not significant.*

### Power Spectral Density

The results of the rPSD comparison between PD and HC groups at rest and during listening to Bach, Mozart, and Dona are described in the following sections and summarized in Table 2. Details on rPSD modifications induced by music compared to rest in the two groups can be found in the Supplementary Material.

**TABLE 2:** Results of EEG power comparison between HC and PD patients.

**Frequency band**	**PSD measure**	**Condition**	**EEG channel**	**PD (*n* = 14)**	**HC (*n* = 12)**	***p*(unc)**	***p*(Bonf)**
Theta	rPSD	Rest	Fp1	14.163	8.442	0.017	n.s.
			T3	15.457	9.912	0.005	0.041
			T4	16.098	10.145	0.019	n.s.
			C3	15.487	9.412	0.019	n.s.
			C4	15.304	8.887	0.025	n.s.
			O1	17.488	8.128	0.015	n.s.
			O2	19.395	7.563	0.025	n.s.
		Dona	Fp1	13.890	9.433	0.025	n.s.
		Mozart	Fp1	14.229	8.453	0.015	n.s.
	rPSD_%__change_	Bach	T3	−2.25	35.82	0.011	n.s.
			O1	−1.01	30.52	0.008	n.s.
			O2	−5.97	28.81	0.020	n.s.
		Dona	T3	7.58	30.94	0.017	n.s.
			O1	2.29	24.73	0.048	n.s.
			O2	−1.45	48.29	0.042	n.s.
		Mozart	O1	−3.12	51.55	<0.001	0.003
			O2	−5.09	50.98	0.029	n.s.
Alpha	rPSD	Rest	Fp1	15.140	6.980	0.042	n.s.
			Fp2	14.990	8.954	0.042	n.s.
			T3	26.776	17.902	0.025	n.s.
	rPSD_%__change_	Bach	T3	−13.89	17.09	0.013	n.s.
			C3	−1.99	16.23	0.040	n.s.
		Dona	T3	2.87	28.21	0.033	n.s.

*Median values are reported. HC, healthy controls; PD, Parkinson’s disease; EEG, electroencephalography; PSD, power spectral density; rPSD, band-relative PSD, expressed as percentage of total PSD; rPSD_%change_, rPSD percent change with respect to rest; p(unc), uncorrected p-value from two-sided Wilcoxon ranksum test; p(Bonf), Bonferroni corrected p-value from two-sided Wilcoxon ranksum test (n = 8); n.s., not significant.*

#### Theta Band

At rest, PD patients showed significantly higher theta rPSD in the left temporal channel (T3) compared to HC (p_Bonf_< 0.05). The enhanced theta rPSD emerged, as a tendency, also in the other EEG channels except from the right frontopolar one (Fp2) (p_unc_< 0.05). In all channels, theta rPSD remained higher in PD patients than in HC also in the music conditions, but without significant group differences except from a trend in the left frontopolar channel (Fp1) during Mozart and Dona (p_unc_< 0.05). Indeed, music elicited a diffused increase in theta rPSD in HC but not in PD patients. The PD *vs.* HC comparison in terms of music effects with respect to resting state showed significant differences in Mozart, inducing a higher, positive rPSD_%__change_ in left occipital channel (O1) in HC compared to PD patients (p_Bonf_ < 0.05). At the trend level, all music conditions enhanced the occipital theta rPSD more in HC than in PD patients (p_unc_< 0.05). The same tendency emerged in the left temporal channel (T3) during Bach and Dona (p_unc_< 0.05).

#### Alpha Band

During both rest and music, there were no significant group differences in alpha rPSD (p_Bonf_< 0.05). Only during rest, PD patients showed a tendency of higher alpha rPSD in bilateral frontopolar and left temporal channels (Fp1/Fp2 and T3) compared to HC (p_unc_< 0.05). This trend was not confirmed in the music conditions, since music produced alpha rPSD changes with respect to rest in HC but not in PD patients. The group comparison of rPSD_%__change_ from rest to music showed enhanced effects of Bach and Dona in HC compared to PD patients. Indeed, the rPSD_%__change_ induced by (i) Bach in left centrotemporal channels (T3 and C3), (ii) Dona in left temporal channel (T3) were slightly higher in HC than in PD patients (p_unc_< 0.05).

### Granger Causality Analysis

**FIGURE 1:**
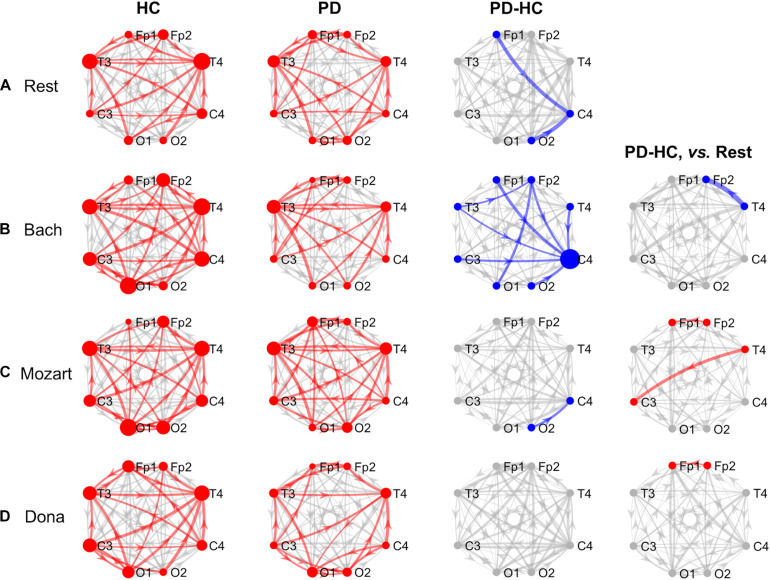
GCA results, theta band. Theta-band information pathways characterizing each group (“HC” and “PD” columns) and their difference between the two groups (“PD-HC” column) during rest (row “**A**”) and music listening (“**B**”–“**D**” rows). “PD-HC, vs. Rest” column: differences between PD and HC concerning the information flow changes induced by the music tracks (“**B**”–“**D**” rows) with respect to rest. In “HC” and “PD” columns, the red links/nodes represent the connections with PDC values significantly higher than the group-level reference PDC value (median of PDC values in the network and across subjects of the group) based on one-sided Wilcoxon ranksum test (*p* < 0.05). In “PD-HC” column, the blue links/nodes highlight the connections with significantly lower PDC values in PD patients than in HC (*p* < 0.05, Bonferroni corrected, *n* = 56). In “PD-HC, vs. Rest” column, red and blue links/nodes highlight the connections with significantly higher and lower PDC_%__change_ values in PD patients than in HC (*p* < 0.05). In all graphs, the link width is proportional to the connection strength (in “PD-HC” columns, its group difference). The node radius is proportional to the sum of nodal inflow and outflow (in “PD-HC” columns, their group difference). GCA, Granger Causality Analysis; PDC, Partial Directed Coherence; PDC_%__change_, PDC percent change with respect to rest; HC, healthy controls; PD, Parkinson’s disease.

**FIGURE 2:**
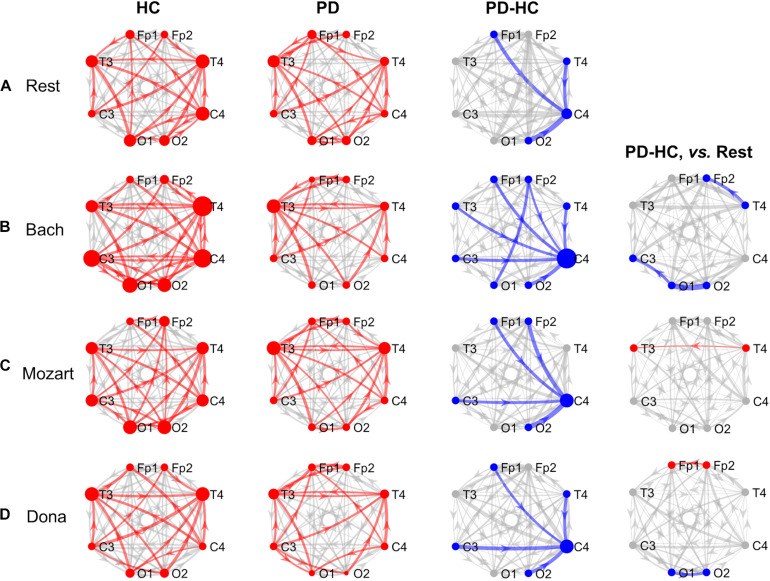
GCA results, alpha band. Alpha-band information pathways characterizing each group (“HC” and “PD” columns) and their difference between the two groups (“PD-HC” column) during rest (row “**A**”) and music listening (“**B**”–“**D**” rows). “PD-HC, vs. Rest” column: differences between PD and HC concerning the information flow changes induced by the music tracks (“**B**”–“**D**” rows) with respect to rest. In “HC” and “PD” columns, the red links/nodes represent the connections with PDC values significantly higher than the group-level reference PDC value (median of PDC values in the network and across subjects of the group) based on one-sided Wilcoxon ranksum test (*p* < 0.05). In “PD-HC” column, the blue links/nodes highlight the connections with significantly lower PDC values in PD patients than in HC (*p* < 0.05, Bonferroni corrected, *n* = 56). In “PD-HC, vs. Rest” column, red and blue links/nodes highlight the connections with significantly higher and lower PDC_%__change_ values in PD patients than in HC (*p* < 0.05). In all graphs, the link width is proportional to the connection strength (in “PD-HC” columns, its group difference). The node radius is proportional to the sum of nodal inflow and outflow (in “PD-HC” columns, their group difference). GCA, Granger Causality Analysis; PDC, Partial Directed Coherence; PDC_%__change_, PDC percent change with respect to rest; HC, healthy controls; PD, Parkinson’s disease.

The effective connectivity patterns characterizing PD and HC groups in the four conditions are described in the following sections. The information pathways characterizing each group and with group differences (p_Bonf_< 0.05) are illustrated in Figure 1 (theta) and Figure 2 (alpha). For each group, the results of the comparison among the EEG nodes based on inflow and outflow are reported in Table 3. The results of the node- and link-level PDC comparisons between PD and HC groups are summarized in Tables 4, 5, respectively. PDC modifications induced by music compared to rest in each group are reported in the Supplementary Material.

**TABLE 3:** Results of intra-group comparison among the EEG nodes in terms of PDC inflow and outflow.

**Frequency band**	**Condition**	**Nodal measure**	**HC**	**PD**
Theta	Rest	Inflow	KW test: *p* < 0.001 *Post-hoc*tests: (1) T4 > C3, C4, O2 (2) T3 > O2	KW test: *p* < 0.001 *Post-hoc*tests: (1) C4 < Fp1, T3, T4, O1, O2
		Outflow	KW test: n.s.	KW test: *p* < 0.001 *Post-hoc*tests: (1) C4 > Fp1, T3 and T4
	Bach	Inflow	KW test: *p* < 0.05 *Post-hoc*tests: (1) T4 > C3	KW test: *p* < 10^–9^ *Post-hoc*tests: (1) C4 < Fp1, T3, T4, O1, O2 (2) Fp2 < Fp1, T3, T4 (3) C3 < T3
		Outflow	KW test: n.s.	KW test: *p* < 0.001 *Post-hoc*tests: (1) C4 > Fp1, T3 and T4 (2) Fp2 > T3
	Mozart	Inflow	KW test: *p* < 0.001 *Post-hoc*tests: (1) T4 > C3, C4, O1, O2	KW test: *p* < 10^–8^ *Post-hoc*tests: (1) C4 < Fp1, T3, T4, O1, O2 (2) Fp2 < Fp1, T3, T4
		Outflow	KW test: *p* < 0.01 *Post-hoc*tests: n.s.	KW test: *p* < 0.01 *Post-hoc*tests: (1) C4 > Fp1, T3 (2) Fp2 > Fp1
	Dona	Inflow	KW test: *p* < 0.01 *Post-hoc*tests: (1) T4 > C3, C4, O1, O2	KW test: *p* < 10^–8^ *Post-hoc*tests: (1) C4 < Fp1, T3, T4, O1, O2 (2) Fp2 < Fp1, T3, T4
		Outflow	KW test: n.s.	KW test: *p* < 0.001 *Post-hoc*tests: (1) C4 > Fp1, T4
Alpha	Rest	Inflow	KW test: *p* < 0.01 *Post-hoc*tests: (1) T4 > C3, O2	KW test: *p* < 10^–9^ *Post-hoc*tests: (1) C4 < Fp1, T3, T4, O1, O2 (2) C3, Fp2 < T3, T4
		Outflow	KW test: *p* < 0.01 *Post-hoc*tests: n.s.	KW test: *p* = 0.001 *Post-hoc*tests: (1) C4 > Fp1, T3, T4
	Bach	Inflow	KW test: *p* < 0.05 *Post-hoc*tests: n.s.	KW test: *p* < 10^–8^ *Post-hoc*tests: (1) C4 < Fp1, T3, T4, O2 (2) Fp2 < Fp1, T3, T4 (3) C3 < T3
		Outflow	KW test: *p* < 0.01 *Post-hoc*tests: n.s.	KW test: *p* < 0.01 *Post-hoc*tests: (1) Fp1 < Fp2, C3, C4
	Mozart	Inflow	KW test: *p* = 0.001 *Post-hoc*tests: (1) T4 > C3, C4, O1, O2	KW test: p < 10^–7^ *Post-hoc*tests: (1) C4 < Fp1, T3, T4, O2 (2) Fp2 < Fp1, T3, T4
		Outflow	KW test: *p* < 0.001 *Post-hoc*tests: (1) O1 > Fp2, T4	KW test: *p* < 10^–4^ *Post-hoc*tests: (1) C4 > Fp1, T3, T4 (2) Fp1 < C3, C4, O1
	Dona	Inflow	KW test: *p* < 0.001 *Post-hoc*tests: (1) T4 > C3, O1, O2	KW test: *p* < 10^–8^ *Post-hoc*tests: (1) C4 < Fp1, T3, T4, O1, O2 (2) Fp2 < Fp1, T3, T4 (3) C3 < Fp1
		Outflow	KW test: *p* < 0.01 *Post-hoc*tests: (1) C3, C4 > Fp1	KW test: *p* < 10^–4^ *Post-hoc*tests: (1) Fp2, C3, C4, O1 > Fp1 (2) C4 > T4

*Median values are reported. PDC, partial directed coherence; HC, healthy controls; PD, Parkinson’s disease; KW, Kruskal-Wallis test; p, p-value; Post-hoc tests, pairwise comparison results (p < 0.05).*

**TABLE 4:** Results of node-level PDC comparisons between HC and PD patients.

**Frequency band**	**Condition**	**Nodal measure**	**EEG node**	**PD (*n* = 14)**	**HC (*n* = 12)**	***p*(unc)**	***p*(Bonf)**
Theta	Rest	Inflow	C4	1.098	2.285	<0.0001	<0.001
	Bach	Inflow	Fp2	1.442	2.915	<0.001	0.007
		Inflow	C4	1.054	2.777	<0.0001	<0.001
		Outflow	T3	1.963	2.755	0.005	0.041
		outflow	C3	2.513	3.218	0.004	0.035
	Mozart	Inflow	C4	0.946	2.495	<0.001	0.002
	Dona	Inflow	C4	1.192	2.096	<0.001	0.003
		Outflow	Fp1	1.786	2.503	0.004	0.035
Alpha	Rest	Inflow	C4	0.989	2.284	<0.0001	0.001
	Bach	Inflow	C4	1.072	3.168	<0.0001	<0.001
		Inflow	Fp2	1.727	3.097	<0.001	0.006
	Mozart	Inflow	C4	0.903	2.575	<0.0001	<0.001
	Dona	Inflow	C4	0.960	2.620	<0.0001	<0.001
		Outflow	Fp1	1.668	2.170	<0.001	0.003

*Median values are reported. PDC, partial directed coherence; HC, healthy controls; PD, Parkinson’s disease; EEG, electroencephalography; p(unc), uncorrected p-value from two-sided Wilcoxon ranksum test; p(Bonf), Bonferroni corrected p-value from two-sided Wilcoxon ranksum test (n = 8).*

**TABLE 5:** Results of link-level PDC comparisons between HC and PD patients.

**Frequency band**	**Condition**	**PDC measure**	**Network link**	**PD**	**HC**	***p*(unc)**	***p*(Bonf)**
Theta	Rest	PDC	Fp1 → C4	0.101	0.301	<0.0001	0.003
			O2 → C4	0.161	0.399	<0.001	0.007
	Bach	PDC	T3 → Fp2	0.186	0.380	<0.001	0.029
			O1 → Fp2	0.186	0.461	<0.001	0.035
			Fp1 → C4	0.099	0.420	<0.001	0.013
			Fp2 → C4	0.140	0.399	<0.001	0.006
			T3 → C4	0.129	0.310	<0.0001	0.005
			T4 → C4	0.150	0.406	<0.001	0.042
			C3 → C4	0.174	0.413	<0.0001	<0.001
			O2 → C4	0.117	0.405	<0.001	0.042
		PDC_%__change_	T4 → Fp2	23.85	62.88	0.035	n.s.
	Mozart	PDC	O2 → C4	0.185	0.398	<0.0001	0.035
		PDC_%__change_	Fp2 → Fp1	19.26	−17.21	0.0168	n.s.
			T4 → C3	21.91	−38.60	0.0253	n.s.
	Dona	PDC_%__change_	Fp2 → Fp1	23.15	−6.22	0.0288	n.s.
Alpha	Rest	PDC	Fp1 → C4	0.117	0.319	<0.001	0.006
			T4 → C4	0.117	0.303	<0.001	0.029
			O2 → C4	0.137	0.418	<0.001	0.011
	Bach	PDC	O1 → Fp2	0.181	0.456	<0.001	0.024
			Fp1 → C4	0.109	0.393	<0.001	0.020
			Fp2 → C4	0.152	0.367	<0.001	0.042
			T3 → C4	0.125	0.377	<0.001	0.007
			T4 → C4	0.138	0.411	<0.001	0.009
			C3 → C4	0.208	0.500	<0.0001	<0.001
			O2 → C4	0.169	0.519	<0.001	0.020
		PDC_%__change_	T4 → Fp2	−21.25	23.49	0.035	n.s.
			O1 → C3	−12.35	27.70	0.023	n.s.
			O1 → O2	−24.28	51.90	0.001	n.s.
	Mozart	PDC	Fp1 → C4	0.076	0.260	<0.001	0.009
			Fp2 → C4	0.118	0.348	<0.001	0.020
			C3 → C4	0.161	0.338	<0.001	0.042
			O2 → C4	0.173	0.466	<0.001	0.020
		PDC_%__change_	T4 → T3	−11.87	−30.49	0.0422	n.s.
	Dona	PDC	Fp1 → C4	0.092	0.282	<0.001	0.035
			T4 → C4	0.108	0.343	<0.001	0.013
			C3 → C4	0.156	0.439	<0.001	0.020
			O2 → C4	0.117	0.390	<0.001	0.024
		PDC_%__change_	O1 → O2	−12.01	34.23	0.019	n.s.
			Fp2 → Fp1	33.07	4.82	0.042	n.s.

*Median values are reported. PDC, partial directed coherence; PDC_%change_, PDC percent change with respect to rest; HC, healthy controls; PD, Parkinson’s disease; EEG, electroencephalography; p(unc), uncorrected p-value from two-sided Wilcoxon ranksum test; p(Bonf), Bonferroni corrected p-value from two-sided Wilcoxon ranksum test (n = 8).*

#### Theta Band and Rest

During rest, HC and PD groups showed both common and distinct patterns of theta PDC information (Figure 1A). The network-level theta PDC strength was slightly higher in HC than in PD patients but comparable (*p* = 0.19, *z* = 1.31). Concerning nodal ranking (Table 3), in both groups the EEG nodes differed in terms of inflow, with the temporal channels being the main network receivers. The nodes with the lowest inflow were in the right occipital region (O2) in HC, and in the right central region (C4) in PD patients. The EEG nodes showed significant outflow differences only in the PD group, with C4 emerging as the main network source. The node- and link-level PDC group comparisons (Tables 4, 5) showed deficits of information entering in C4, specifically from the left frontopolar and right occipital channels (Fp1 and O2), in PD patients compared to HC (p_Bonf_< 0.05). The two groups did not differ in terms of nodal outflows.

#### Theta Band and Music

During music listening, the network-level theta PDC strength remained higher in HC than in PD patients. The PDC differences between groups largely depended on the music track: the connectivity patterns during Dona were comparable between groups, whereas Bach was associated with widespread PDC differences. In all music conditions, in both HC and PD groups, the main receivers of theta information remained the temporal channels. No clear sources of information emerged in the EEG network of HC, whereas the right central and frontopolar channels (C4 and Fp2) emerged as key information sources in PD patients. In PD, music enhanced the frontotemporal inter-hemispheric communication, but was not able to compensate the right central (C4) inflow deficits in comparison with HC. The results concerning the single music tracks are reported hereinafter.

##### Bach

During Bach listening (Figure 1B), the network-level theta PDC strength was significantly higher in HC than in PD patients (*p* = 0.007, *z* = 2.70). As detailed in Table 3, HC were characterized by slight inflow differences among the EEG nodes, with the right temporal channel (T4) being the main receiver. PD patients showed highly significant inflow differences among the EEG nodes, with the central, especially in the right side (C4), and right frontopolar (Fp2) channels receiving lower information than the others, especially temporal ones. PD patients but not HC were characterized by differences among the EEG nodes in terms of outflow, with the maximum information exiting from C4, followed by Fp2.

The node-level PDC comparison between the two groups (Table 4) showed (i) lower inflow in right frontopolar and central channels (Fp2 and C4), (ii) lower outflow from left centrotemporal channels (T3 and C3) in PD compared to HC (p_Bonf_< 0.05). Accordingly, in PD patients, the link-level PDC comparison (Table 5) showed deficits of information (i) from all EEG nodes except the left occipital one (O1) to the right central channel (C4), and (ii) from the left temporooccipital channels (T3 and O1) to the right frontopolar one (Fp2) (p_Bonf_< 0.05). Group differences were thus enhanced during Bach listening compared to resting-state condition.

The two groups did not differ in terms of PDC_%__change_ from rest to Bach except from the information flow from the right temporal (T4) to the right frontopolar (Fp2) channels, which increased in HC and decreased in PD patients (p_unc_< 0.05) (Table 5). No group differences emerged in terms of node-level PDC_%__change_ from rest to Bach.

##### Mozart

During Mozart listening (Figure 1C), the network-level theta PDC strength was comparable between groups (*p* = 0.26, *z* = 1.11). As shown in Table 3, HC were characterized by differences in nodal inflow among the EEG nodes, with the right temporal channel (T4) being the main network receiver. Much more significant nodal inflow differences emerged in PD patients, with the temporal channels being the main receivers and the right central and frontopolar channels (C4 and Fp2) being the nodes with lowest incoming information. Both groups showed outflow differences among the network nodes, confirmed at the pairwise level only in PD patients. In this group, the main driver of information was C4, followed by Fp2.

The node-level PD *vs*. HC comparison showed that the right central (C4) inflow was lower in PD patients than in HC (p_Bonf_< 0.05) (Table 4). At the link level, PD patients were characterized by lower information from the right occipital channel (O2) to the homolateral central channel (C4) compared to HC (p_Bonf_ < 0.05) (Table 5). Significant group differences emerged in the PDC_%__change_ from rest to Mozart in the frontopolar connections from right to left (from Fp2 to Fp1) and from the right temporal (T4) to the left central (C3) channels, being negative in HC and positive in PD patients (p_unc_< 0.05) (Table 5). No group differences emerged in node-level PDC_%__change_ values.

##### Dona

During Dona listening (Figure 1D), the two groups were comparable in terms of network-level theta PDC strength (*p* = 0.34, *z* = 0.95). Both groups were characterized by significant differences among the EEG nodes in terms of inflow (Table 3), with the right temporal channel (T4) as the main network receiver. Only in PD patients, the right central and frontopolar (C4 and Fp2) channels emerged for receiving less information than the others. Significant outflow differences among the EEG nodes emerged only in the PD group, with C4 being the main information source.

The group comparison showed differences only at the node level, with PD patients characterized by lower right central (C4) inflow and left frontopolar (Fp1) outflow than HC (p_Bonf_< 0.05) (Table 4). PD and HC groups were comparable in the link-level PDC patterns. The groups differed in the PDC_%__change_ from rest to Dona in the frontopolar connections from right to left (from Fp2 to Fp1), being negative in HC and positive in PD patients (p_unc_< 0.05) (Table 5). No group differences in node-level PDC_%__change_ values emerged.

#### Alpha Band and Rest

The alpha connectivity patterns emerged during rest are shown in Figure 2A. The network-level alpha PDC strength was slightly higher in HC than in PD patients but comparable (*p* = 0.14, *z* = 1.47). Concerning nodal ranking (Table 3), both groups showed inflow differences among the EEG nodes, with higher significance in PD patients than in HC. Like in theta, the main network receivers were the temporal channels, especially the right one (T4). The lowest information was received by the right occipital node (O2) in HC, the right central node (C4) in PD patients. Significant outflow differences emerged among the EEG nodes in both HC and PD patients. In both groups, the channel with highest outflow was the right central one (C4).

The node-level comparison between groups showed inflow deficits in C4 in PD compared to HC (p_Bonf_< 0.05) (Table 4). At the link level, PD patients were characterized by lower PDC from left frontopolar (Fp1) and right temporooccipital (T4 and O2) channels to the right centralone (C4) compared to HC (p_Bonf_< 0.05) (Table 5).

#### Alpha Band and Music

In all music conditions, the network-level alpha PDC strength remained higher in HC than in PD patients. As in theta, the highest alpha PDC differences between HC and PD were observed during Bach listening. During music listening, as in resting state, the temporal channels usually received more information than the others, with some exceptions in the PD group, where also the left frontopolar channel (Fp1) was a key receiver. Accordingly, the central channels remained the major network sources (overcome only by occipital channels in HC while listening to Mozart). In PD patients, music elicited the inter-hemispheric frontal communication with respect to rest but did not compensate for the right central (C4) inflow deficits characterizing PD patients compared to HC. The results concerning the single music conditions are reported hereinafter.

##### Bach

During Bach listening (Figure 2B), the network-level alpha PDC strength was significantly higher in HC than in PD patients (*p* = 0.003, *z* = 2.96). As detailed in Table 3, HC and PD patients showed slight and highly significant inflow differences among the EEG nodes, respectively. The main network receivers were the right (T4) and left (T3) temporal channels in HC and PD patients, respectively. In PD patients, the right central and frontal channels received less information than other channels in the network. Slight outflow differences among the EEG nodes emerged in both groups, with the right central (C4) and left frontopolar (Fp1) ones having the highest and lowest outflow values, respectively.

The node-level group PDC comparison showed significant differences in the right frontopolar and central (Fp2 and C4) inflows, being lower in PD patients compared to HC (p_Bonf_< 0.05) (Table 4). At the link level, PD patients were characterized by lower PDC from the left occipital node (O1) to the right frontopolar node (Fp2), and from all channels but O1 to the right central one (C4) (p_Bonf_< 0.05) (Table 5). Regarding the PDC changes induced by Bach listening with respect to rest, group differences emerged only at the link-level and regarded the information from the right temporal channel (T4) to the homolateral frontopolar one (Fp2) and from the left occipital channel (O1) to the contralateral and left central ones (C3 and O2), which increased in HC but decreased in PD patients (p_unc_< 0.05) (Table 5).

##### Mozart

During Mozart listening (Figure 2C), the network-level alpha PDC strength was comparable between groups (*p* = 0.17, *z* = 1.36). Significant inflow differences among the EEG nodes emerged in both HC and PD patients (Table 3). The main network receiver was the right temporal node (T4) in HC, together with the left temporal and frontopolar nodes (T3 and Fp1) in PD patients. In PD patients, the right central channel (C4) received the minimum amount of information, followed by the homolateral frontopolar channel (Fp2). Significant outflow differences among the EEG nodes emerged in both groups. The main sources of information in HC and PD patients were in left occipital (O1) and right central (C4) nodes, respectively. In PD patients, the left frontopolar channel (Fp1) sent the minimum amount of information.

The node-level group PDC comparison showed significant right central (C4) inflow deficits in PD patients compared to HC (p_Bonf_< 0.05) (Table 4). At the link level, PD patients received less information than HC from the frontopolar (Fp1/2), left central (C3) and right occipital (O2) channels to the right central one (C4) (p_Bonf_< 0.05) (Table 5). With respect to rest, Mozart listening induced a more negative PDC_%__change_ in HC compared to PD patients in the temporal connections from right to left (from T4 to T3) (*p* < 0.05) (Table 5).

##### Dona

During Dona listening (Figure 2D), the network-level alpha PDC strength was comparable between HC and PD groups (*p* = 0.10, *z* = 1.67). As listed in Table 3, significant inflow differences among the EEG nodes emerged in both groups. In HC, the main receiver was the right temporal channel T4, which in PD patients was preceded by the left frontopolar one (Fp1). The right frontopolar and central channels (Fp2 and C4) received the lowest amount of information from the network. Significant outflow differences emerged among the EEG nodes, especially in PD. In both groups, the central nodes were the main information sources, and the left frontopolar (Fp1) was the node with lowest outflow.

Both node-level and link-level PDC differences emerged between the two groups. PD patients were characterized by lower inflow in the right central (C4) and lower outflow in left frontopolar (Fp1) nodes (p_Bonf_< 0.05) (Table 4). Specifically, C4 received less information from the left frontopolar (Fp1) and central (C3) and the right temporooccipital (T4 and O2) nodes in PD patients compared to HC (p_Bonf_< 0.05) (Table 5). The HC *vs.* PD comparison based on PDC_%__change_ from rest to Dona showed significant differences in the information (i) from left to right in the occipital area (from O1 to O2), being positive in HC and negative in PD patients, and (ii) from right to left in the frontopolar area (from Fp2 to Fp1), being much more positive in the PD group (p_unc_< 0.05) (Table 5).

## Discussion

In this EEG study, for the first time, we combined directional connectivity and power spectral analyses to investigate the neurophysiological correlates of PD, both at rest and while listening to different music themes. A primary objective was to examine, using GCA, the basal pathways of propagation of information within the brain network of PD patients in comparison with healthy elders. Then, in view of the proven beneficial effects of music-based interventions on PD (Garcia-Casares et al., 2018), we were interested in exploring whether and how music listening could modify the brain connectivity patterns. Traditional EEG power analyses were also conducted to frame the GCA results and enrich their interpretation.

Our results demonstrate the importance of examining the dynamical relations among brain regions in the study of the neural bases of PD. Indeed, while the rPSD comparison showed enhanced theta power and slightly enhanced alpha power in PD patients compared to HC, especially at rest, the GCA comparison revealed marked deficits of theta and alpha communication in the EEG network of PD patients. These opposite tendencies suggest that excess of EEG power observed in PD patients might correspond to reduced interactions among the EEG network nodes.

For the first time we found that, regardless of the experimental condition, the theta and alpha information received by the right central channel (C4) from the rest of the EEG network was significantly lower in PD patients than in HC. This robust finding suggests a peculiar role of the right sensorimotor cortex in PD pathophysiology, opening up new avenues in the search for brain network underpinnings of PD and in the identification of more targeted therapeutic pathways.

Compared to traditional power analyses, the GCA approach provided additional, valuable information on the effects of music on brain processes in PD patients. Indeed, while music produced significant increases in theta and alpha EEG power in the HC group, it induced only modest, non-significant EEG power changes in the PD group. On the contrary, GCA revealed that music produced remarkable changes in the connectivity patterns of PD patients, enhancing the frontotemporal inter-hemispheric communication with respect to rest. Still, music was not able to compensate for the connectivity deficits in the right central channel characterizing PD patients.

Our findings also suggest that brain responses to music largely depend on the music-specific characteristics, possibly reflecting the melodic structure, musical complexity and induced attentional and emotional states. The maximum group connectivity differences emerged during Bach listening, whereas the Dona listening generated the most comparable connectivity patterns between PD patients and HC. When compared to rest, Bach induced more widespread intra- and inter-hemispheric communication in HC than in PD patients. Conversely, the effects of Mozart and Dona were enhanced in PD patients. This evidence, besides being preliminary, might route the selection of *ad-hoc* rhythmic sound stimulations toward more effective music-based interventions for PD.

### Background EEG in PD

In resting state, we found a diffused increase in theta power and slightly enhanced frontal alpha power in the patient group. Our findings support the already robust literature evidence on PD, showing excessive EEG/MEG power in the low frequency range in comparison with HC (Bosboom et al., 2006; Stoffers et al., 2007; Moazami-Goudarzi et al., 2008). The slowing of brain electrical activity was hypothesized to be an intrinsic characteristic of the parkinsonian population, already present in the early illness stages and largely unrelated to pharmacological medication as well as duration, stage and severity of the disease (Stoffers et al., 2007). Other research works suggested that the amount EEG slowing could be proportional to cognitive impairment and predict dementia development in PD patients (Guner et al., 2017). Longitudinal studies support this theory, showing an association between decreasing cognitive performance and low frequency power increases over time (Olde Dubbelink et al., 2013). In our PD population, the MOCA and MMSE scores were in the normal range (Conti et al., 2015; Santangelo et al., 2015) and comparable to healthy elders. Nevertheless, it would be interesting to check whether the increase in theta power in our non-demented PD patients is predictive of mild cognitive impairment (MCI) or dementia.

The GCA comparison confirmed that the EEG power abnormalities in our PD sample were associated with altered interactions within the EEG network at rest. Indeed, PD patients showed slightly lower global connectivity than HC. This finding is supported by recent fMRI evidence of reduced information flow among motor-related brain regions in PD (Wu et al., 2012b; Ghasemi and Mahloojifar, 2013). Notably, a marked difference emerged in the role of the right central channel, which in the PD network was the main source of information but showed a deficient role as receiver. This finding confirms that PD pathophysiology involves altered interactions of the sensorimotor cortex with other brain regions, and further suggests a right-dominance of these alterations that seems to be independent on the laterality of motor symptoms.

To our knowledge, our study is the first to explore EEG-based effective connectivity patterns in PD patients at rest. Our results extend previous evidence of altered directional connectivity in PD during movement preparation and execution (Tropini et al., 2011), confirming the existence of these abnormalities even in the background EEG rhythms. Our findings are supported by resting-state fMRI evidence of smaller inflow in the motor regions (Ghasemi and Mahloojifar, 2013) and smaller flow from the substantianigra pars compacta to the supplementary motor area (Wu et al., 2012b). Contrasting findings emerged from a recent fMRI study, reporting an increased flow of information from the left premotor cortex to the right primary motor region (Hao et al., 2020).

Our GCA evidence may suggest that spontaneous mental processes are differently lateralized in PD patients compared to healthy elders. Of note, this lateralization was not observed in our EEG power analysis; in the same line, a previous EEG power study showed lateralized frontal activity but in the theta band, mainly associated with clinical disability (Mostile et al., 2015). Most studies on lateralization of brain function in PD have been performed using fMRI, providing results that are not in conflict with ours. Abnormalities in the right hemisphere were highlighted by Huang et al., who proposed that right frontoparietal connectivity could be used as candidate biomarker for PD (Huang et al., 2019). Another fMRI study showed abnormal inter-hemispheric connections between homotopic sensorimotor regions in PD patients, suggesting deficient cooperation between these two brain areas that might contribute to motor impairment (Luo et al., 2015).

### Music Effects on EEG Activity

Our EEG power analyses showed that the differences between PD patients and HC observed at rest were mitigated by the three music conditions, which enhanced the EEG power much more in HC than in PD. HC showed a general increase in theta activity, especially in the left occipital lobe, and enhanced alpha activity in the left central and temporal regions. In HC, music effects on EEG power depended on the music track. Curiously, Bach and Dona but not Mozart enhanced the alpha rhythm, while theta power changes resulted more significant during Mozart and Bach listening.

Up until now, the study of music effects on the brain electrical activity has primarily focused on pieces of music from Mozart, which were shown to exert a positive influence on various neurocognitive domains. Different research works reported a temporary enhancement of spatial-temporal reasoning performance after listening to Mozart’s music, giving rise to the so-called “Mozart Effect” (Rauscher et al., 1993; Jenkins, 2001). Such effect was firstly referred to the Sonata in D Major for Two Pianos K448, which showed beneficial influences in HC and in subjects with MCI (Cacciafesta et al., 2010) and epilepsy (Hughes et al., 1998). Nevertheless, further investigations either failed to demonstrate the Mozart effect (Chabris, 1999) or suggested that it might be shared by compositions similar in tempo, structure, melody and harmony (Hughes and Fino, 2000).

The search for the neural bases of music perception, which is needed to understand its positive effects, is still in its infancy. The EEG literature showed alpha power increase and alpha complexity decrease in HC after listening to the Mozart’s K448 Sonata (Jausovec et al., 2006; Verrusio et al., 2015). Although these specific results were not replicated with Beethoven (Verrusio et al., 2015) or Brahms (Jausovec et al., 2006), there is evidence of an involvement of parieto-occipital alpha rhythm during both perception and imagination of natural musical phrases (Schaefer et al., 2011). Our results extend this evidence by showing that music-induced EEG power changes were partially shared across the three music themes, especially in the theta band. The shared increase of theta power in occipital cortex and left temporal cortex might result from the listening to natural music in general. The less pronounced alpha power changes might be the consequence of the progressive slowdown of the EEG activity occurring with aging processes (Klass and Brenner, 1995) and/or the dominance of the alpha rhythm during the resting state. In this line, the observation of alpha power increases in the central and temporal regions but not in the occipital one might be explained by the prevalence of the occipital alpha activity at rest. Curiously, in our HC group the minimum effects on the alpha rhythm were exerted by the Mozart’s composition, which however differed from the most commonly used K448 sonata. Due to the hypothesized involvement of frontocentral regions in music-induced emotional valence and arousal, emotion-dependent EEG activity may have introduced heterogeneity and affected our group-level EEG results (Lin et al., 2014).

The lack of significant music effects on the EEG power in our patients represents a novel evidence that, if confirmed, can give rise to several hypotheses on PD pathophysiology. The enhanced background EEG activity characterizing PD patients compared to HC might partially explain this result. Of note, similar results emerged in subjects with MCI (Verrusio et al., 2015), which is known to affect a non-negligible portion of PD patients (Cammisuli et al., 2019). Notably, music-induced EEG power increase, but in the gamma band, was found to be lower in MCI than in HC, but much higher in individuals with Alzheimer’s disease (van Deursen et al., 2008). Further studies are needed to test if this evidence is an unspecific consequence of neurodegenerative mechanisms, or if it derives from a deficient processing of rhythmic patterns that might affect not only music perception, but also movement and coordination. Indeed, a large body of literature evidence has shown that auditory stimuli like music can modulate the activity of the brain motor pathways (Koshimori and Thaut, 2018).

### Music Effects on EEG Connectivity

Our EEG study has been the first to investigate the effects of music listening on cerebral effective connectivity in PD patients. In contrast with the EEG power results, the connectivity abnormalities observed in PD patients at rest were either slightly or not compensated by music, whose effects largely depended on the composition. In all music conditions, the node-level inflow deficits in the right central channel remained significant. This important finding might indicate that simple music listening was not able to entirely compensate for the abnormal sensorimotor connectivity characterizing PD patients at rest. Since the brain areas involving rhythm perception and movement regulation are closely related, we cannot exclude that music-based training might modify such response in the long term, facilitating sequential movements and mitigating the lack of dopaminergic stimulation that characterizes PD (Raglio, 2015).

Nevertheless, Dona and Mozart (but not Bach) listening exerted “normalizing” effects on the global information flow in PD, which resulted more similar than in resting state and comparable between PD patients and HC. The highest compensatory effect was exerted by the Dona composition in the theta band, which produced a comparable link-level flow of information between PD and HC groups.

Curiously, the differences in the theta and alpha effective connectivity patterns between PD patients and HC were even exacerbated during Bach listening. This evidence might be explained by the peculiar musical aspects characterizing Bach’s sonata. Bach was defined as a “musical master of mathematical manipulation” for his compositional techniques. Since the distribution of melodic intervals in some of his masterpieces follows the self-similarity rule of fractal structures (Brothers, 2010), the reduced connectivity changes observed in PD during Bach listening might be associated with the deficient chaotic dynamics characterizing the EEG of PD patients compared to HC (Stam et al., 1994). The EEG connectivity patterns might reflect the emotions conveyed by music as well; indeed, a previous study exploring music-related PDC changes showed that, in healthy subjects, pleasant music induces higher theta synchronization with respect to unpleasant music (Varotto et al., 2012).

Despite the just mentioned theme-specific effects, an interesting finding is that music elicited the frontotemporal inter-hemispheric connectivity, from the right side to the left side, in PD patients. In Dona and Mozart, the increase in theta connectivity from Fp2 to Fp1 was significantly higher in patients than in HC. On the contrary, Bach and Dona elicited an increase in left-to-right alpha occipital connectivity that was higher in HC than in PD patients. The enhancement of alpha occipital connections was already reported during music perception (Wu et al., 2012a). Frontotemporal communication is involved in the recognition of emotions from music (Omar et al., 2011), which is topic of research in PD (Wu et al., 2019), and was found to be impaired during motor-language processing in PD. Since music-induced interactions among frontal, auditory and motor regions underlie motor coordination in response to a rhythm (Large et al., 2015), the altered connectivity pattern observed in PD patients while listening to music could reflect to some extent their compromised motor coordination. Overall, our results confirm the ability of music to modulate the brain networks involved in emotional, cognitive and movement regulation, and suggest that an effective rehabilitation of motor functions in PD might be obtained by acting on the underlying neuronal synchronization mechanisms through music therapy.

### Study Limitations, Future Perspectives, and Clinical Relevance

A major limitation of our study concerns the modest sample size, which limited the statistical power of the results and should be enlarged in future studies in order to verify their reproducibility. The reliability and specificity of our findings might also be affected by the patients’ pharmacological therapy. In this respect, future investigations should include naïve patients or analyze EEG power and connectivity both before and after levodopa administration. Further limitations are the low spatial resolution of the EEG results, which could be overcome by using an EEG cap with a higher number of electrodes, and the non-consideration of possible effects of cognition on rest and music-based EEG characteristics. The study results could be extended by (1) considering additional frequency bands and music styles, (2) evaluating the correlations between EEG measures and motor and neuropsychological test scores, (3) characterizing music in terms of dynamic, harmonic, tonal, phonic, and rhythmic features and explore their specific influence on EEG measures (including EEG abnormalities in PD), and (4) performing time-varying music and EEG data analyses. In the future, the adoption of a longitudinal design is also highly encouraged. Follow-up evaluations would allow to monitor how the emerged EEG characteristics evolve as the disease progresses. The repetition of this study after a music-based intervention could clarify the modifications of the neural bases of music perception induced by music therapy in PD. Notably, the majority of subjects from our PD sample is taking part to a music-based neuromotor rehabilitation program, whose effectiveness will be assessed in the near future.

Despite being preliminary, our unprecedented evidence of dysfunctional brain networks underlying rest and music listening in PD individuals has translational potential in the clinics. The emerged communication deficits between the right sensorimotor cortex and the rest of the brain could serve as novel therapeutic target and be used—together with clinical outcomes—to assess the efficacy of pharmacological and non-pharmacological interventions. Furthermore, the music-specific EEG patterns observed in both HC and PD subjects confirm the close link between music features and neuronal oscillations and provide a neurobiological basis for selecting the music themes with highest therapeutic potential. Our preliminary results suggest that Mozart and Dona compositions enhance the brain functional circuits involved in PD and might be preferred to Bach composition in the context of dance/music therapies aimed at recovering functional mobility and balance. The matching among specific music features, EEG patterns and motor, cognitive, and emotional responses will hopefully enable the implementation of optimized user-centered preventive and rehabilitation strategies.

## Conclusion

Although functional brain network abnormalities have been increasingly reported in PD patients, the neural mechanisms underlying the response to innovative therapeutic pathways, including music- or rhythm-based interventions, are still debated. For the first time, we explored the EEG activity and effective connectivity at rest and during music listening in a sample of PD patients and healthy elders. Our results suggest that PD is characterized by enhanced activity but reduced flow of information within the EEG network. The connectivity alterations predominantly involved the right sensorimotor cortex, regardless of the main side of motor symptoms, and were only partially compensated by music listening. Of note, music listening enhanced the frontotemporal connections in PD patients, and thus might modulate the functionality of the brain networks already involved in PD symptomatology. Further studies are needed to ascertain the pathophysiology underlying the emerged EEG abnormalities, verify whether they are influenced by the progression of the disease, and if and how music-based treatment can modulate them.

## Data Availability Statement

The raw data supporting the conclusions of this article will be made available on request by the authors, without undue reservation. Matlab codes are available at the Opens Science Framework web application link: https://osf.io/2h4ws/?view_only=d97c62439933402891ecfd6d02623fd0.

## Ethics Statement

This study involved human participants and was reviewed and approved by the Comitato Etico Milano Area 2, via F. Sforza 28, 20122, Milano, Italy. The patients/participants provided their written informed consent to participate in this study.

## Author Contributions

EM: conceptualization, data curation, methodology, software, and writing—original draft. FA: conceptualization, data curation, and writing—original draft. SM: conceptualization, data curation, and writing—review and editing. FM: data curation and writing—review and editing. LB, FC, and SB: formal analysis, supervision, and writing—review and editing. MN: data curation, methodology, and writing—review and editing. AMB and SC: conceptualization, methodology, and writing—review and editing. PB: supervision and writing—review and editing. GA: conceptualization, formal analysis, project administration, supervision, and writing—review and editing. All authors contributed to the article and approved the submitted version.

## Conflict of Interest

The authors declare that the research was conducted in the absence of any commercial or financial relationships that could be construed as a potential conflict of interest.

## Footnotes

1 https://www.nitrc.org/projects/gmac_2012/
