# Supplementary material for: Effective Connectivity During Rest and Music Listening: An EEG Study on Parkinson’s Disease
Source: Front Aging Neurosci. 2021 Apr 28;13:657221. doi: 10.3389/fnagi.2021.657221 (PMC8113619; doi:10.3389/fnagi.2021.657221)
Supplement: Supplementary file 1 [file Data_Sheet_1.pdf]

# *Supplementary material*

## 1. Supplementary data

The following sections include details on the relative Power Spectral Density (rPSD) and Partial Directed Coherence (PDC) modifications induced by music compared to resting state in Parkinson's disease (PD) patients and healthy controls (HC).

### 1.1 Power spectral density

*Theta band.* Music listening induced significant changes in theta rPSD when compared to resting state in HC but not in PD patients. In HC, Mozart induced a significant positive theta $rPSD_{\%change}$ in O1 ($p_{Bonf} <0.05$) and in T3 and O2 ($p_{unc} <0.05$). Similar results emerged during Bach listening, which induced a positive theta $rPSD_{\%change}$ with respect to rest in C3, T3, O1 and O2 ($p_{unc}<0.05$), being highly significant in O1 ($p_{Bonf} <0.05$). Dona caused an overall increase in theta rPSD that involved most EEG channels (Fp2, T3, C3, C4, O1 and O2), even if at the uncorrected level ($p_{unc} <0.05$).

*Alpha band.* Music listening produced selective alpha rPSD changes with respect to rest in HC but not in PD patients. In HC, Bach and Dona but not Mozart enhanced the alpha rPSD in central and temporal channels of the left hemisphere ($p_{unc} <0.05$). The alpha $rPSD_{\%change}$ induced by Dona in T3 reached a high significance ($p_{Bonf} <0.05$).

### 1.2 Granger causality analysis

When compared to resting state, music listening induced changes in the effective connectivity patterns that varied among the music tracks and between HC and PD.
*Theta band.* Figure S2A, left panel, shows the network links with $PDC_{\%change}$ induced by Bach listening ($p_{unc} <0.05$). In HC, a positive $PDC_{\%change}$ emerged 1) from T4 to Fp2 and O2, 2) from O1 to C3 and O2, whereas a negative $PDC_{\%change}$ emerged from C3 to T3. Of note, the information exiting T3 significantly increased (positive $outflow_{\%change}$) ($p_{unc} <0.05$). In PD patients, a positive $PDC_{\%change}$ emerged only in the connection from T4 to T3.
In the two groups, Mozart listening did not induce significant changes of nodal PDC inflow and outflow with respect to rest, but selective link-level changes emerged (Figure S2B, left panel). In HC, Mozart induced 1) a negative $PDC_{\%change}$ in the connection from T3 to C3, 2) a positive $PDC_{\%change}$ in the connection from O1 to O2. In PD patients, there was an increase of information from (i) Fp2 to Fp1, (ii) T4 to T3 ($p_{unc} <0.05$).
Group-specific node- and link-level PDC changes were induced by Dona compared to rest (Figure S2C, left panel). HC were characterized by a decrease in T3 inflow, specifically in the information from C4 to T3 ($p_{unc} <0.05$). In HC, a positive $PDC_{\%change}$ emerged in the connection from O1 to C3 ($p_{unc} <0.05$). In PD patients, no significant changes in nodal inflow or outflow, but positive $PDC_{\%change}$ emerged in the information from (i) Fp2 to Fp1, and (ii) C3 and T3 to O1 ($p_{unc} <0.05$).

*Alpha band.* In both groups, Bach listening did not induce changes in nodal PDC inflow with respect to rest. Outflow changes emerged in HC but not in PD patients; in HC, Bach induced a significant increase in outflow from the temporal channels ($p_{unc}<0.05$). Selective link-level changes were observed in both groups (Figure S2A, right panel). Negative $PDC_{\%change}$ emerged from C3 to T3 in HC, from C4 to O1 and from Fp1 to T4 in PD patients ($p_{unc}<0.05$). Positive $PDC_{\%change}$ emerged from T4 to T3 and from O1 to O2 in HC, from Fp2 to Fp1 in PD patients ($p_{unc}<0.05$).
Mozart listening did not produce changes in the nodal inflow/outflow with respect to rest. Selective link-level PDC changes emerged in PD patients but not in HC (Figure S2B, right panel). In PD patients, positive $PDC_{\%change}$ from O2 to Fp2 and from Fp2 to Fp1 was accompanied by a negative $PDC_{\%change}$ from Fp1 to T4 ($p_{unc}<0.05$).
Dona listening produced node-level PDC changes only in PD patients, showing a decrease of Fp1 outflow and an increase of O1 inflow ($p_{unc}<0.05$). Group-specific link-level changes emerged (Figure S2C, right panel). In HC, Dona was associated with positive $PDC_{\%change}$ from O1 to O2, negative $PDC_{\%change}$ from C3 to T3 ($p_{unc}<0.05$). In PD patients, Dona induced positive $PDC_{\%change}$ from Fp2 to Fp1 and from C3 to O1, negative $PDC_{\%change}$ from Fp1 to T4 and from O1 to T3 ($p_{unc}<0.05$).

## 2. Supplementary figures

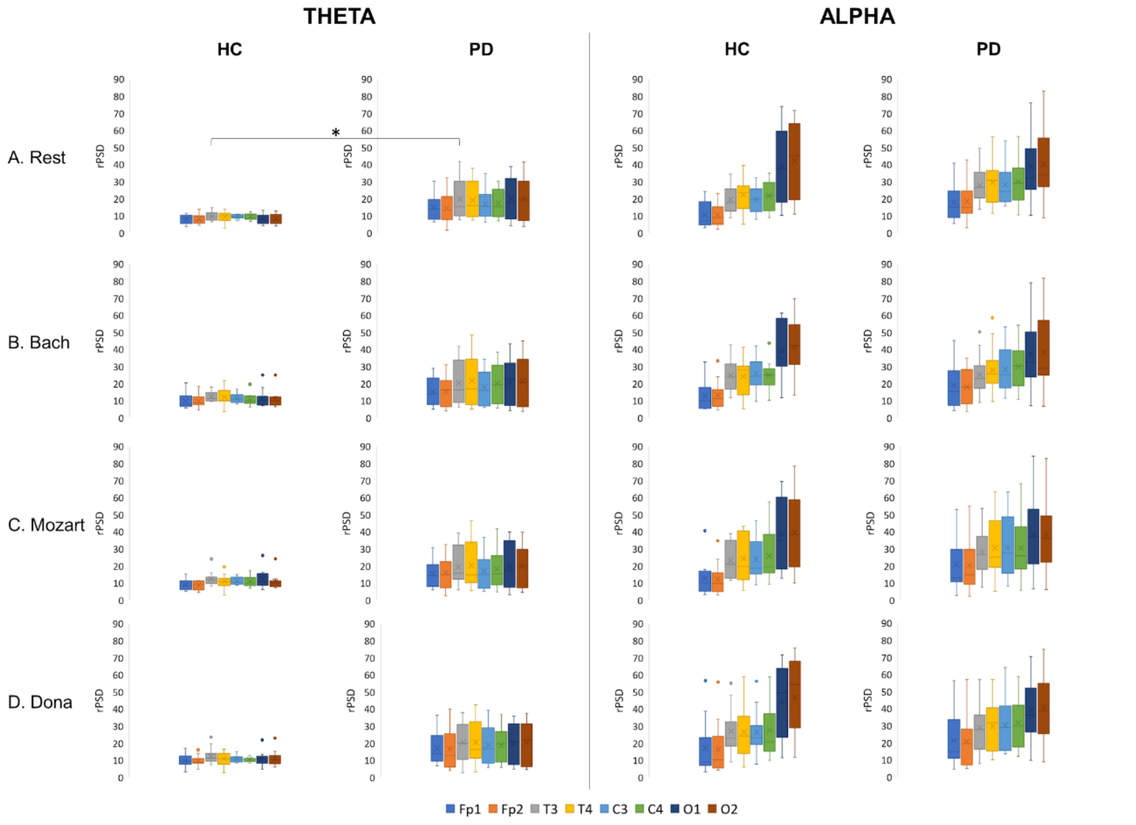


**Supplementary Figure 1. rPSD results.** Box and whisker plots showing the distribution of rPSD values in EEG channels in theta and alpha bands (“Theta” and “Alpha” panels) in HC and PD groups (“HC” and “PD” columns), at rest (“A” row) and during music listening (“B” to “D” rows). Significant group differences are marked with a squared parenthesis and *. rPSD: band-relative power spectral density, expressed as percentage of the total.

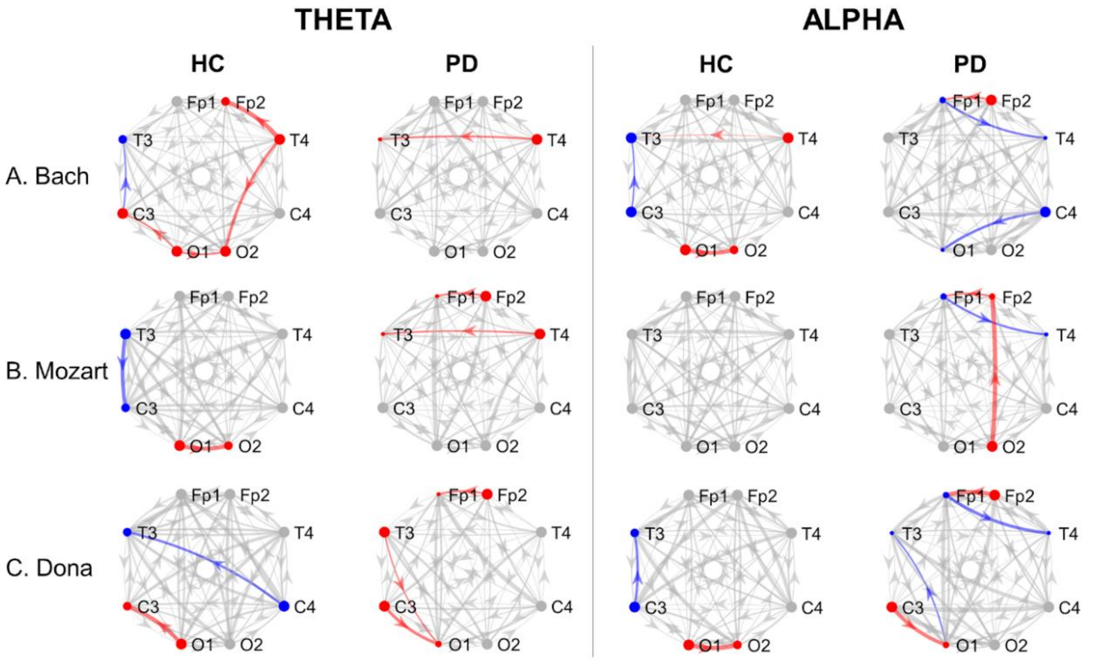


**Supplementary Figure 2. Music effects on PDC values.** Theta-band (left panel) and alpha-band (right panel) information flow changes induced by the three music tracks ( "A" to "C" rows) compared to rest in HC ("HC" column) and PD subjects ("PD" column). Red and blue links/nodes mark the connections with significant positive and negative $PDC_{\%change}$ values from rest to music based on one-sided Wilcoxon signed rank test ($p<0.05$). In all graphs, the link width is proportional to the $PDC_{\%change}$ strength. The node radius is proportional to the sum of nodal inflow and outflow changes. PDC: Partial Directed Coherence. $PDC_{\%change}$: PDC percent change with respect to rest. HC: healthy controls. PD: Parkinson's disease.
